# A mixed reception: perceptions of pregnant adolescents’ experiences with health care workers in Cape Town, South Africa

**DOI:** 10.1186/s12978-021-01211-x

**Published:** 2021-08-04

**Authors:** Ronel Sewpaul, Rik Crutzen, Natisha Dukhi, Derrick Sekgala, Priscilla Reddy

**Affiliations:** 1 Department of Health Promotion, Maastricht University/CAPHRI, Minderbroedersberg 4-6, 6211 LK Maastricht, The Netherlands; 2 Health & Wellbeing, Human and Social Capabilities Division, Human Sciences Research Council, 118 Buitengraght Street, Cape Town, 8000 South Africa; 3 Faculty of Health Sciences, Nelson Mandela University, Port Elizabeth, South Africa

**Keywords:** Teenage pregnancy, Adolescents, Antenatal care, Sexual and reproductive health services, South Africa, Maternal healthcare workers, Antenatal attendance

## Abstract

**Background:**

Maternal mortality among adolescent mothers in South Africa is higher than many middle-income countries. This is largely attributable to conditions that can be prevented or managed by high quality antenatal care. The way in which pregnant adolescents are treated at antenatal clinics influences their timely utilization of antenatal services. This qualitative study reports on the experiences of pregnant adolescents with health care workers when accessing antenatal care.

**Methods:**

Pregnant girls aged 13–19 (n = 19) who attended public health care facilities that provide Basic Antenatal Care (BANC) services in Cape Town, South Africa were recruited. Four face to face in-depth interviews and four mini focus group discussions were undertaken, facilitated by a topic guide. Thematic analyses were used to analyse the data.

**Results:**

Experiences that reinforce antenatal attendance, such as respectful and supportive treatment, were outweighed by negative experiences, such as victimization; discrimination against being pregnant at a young age; experiencing disregard and exclusion; inadequate provision of information about pregnancy, health and childbirth; clinic attendance discouragement; and mental health turmoil.

**Conclusions:**

There is evidence of a discordant relationship between the health care workers and the pregnant adolescents. Adolescents feel mistreated and discriminated against by the health care workers, which in turn discourages their attendance at antenatal clinics. Maternal health care workers need to receive support and regular training on the provision of youth friendly antenatal care and be regularly evaluated, to promote the provision of fair and high quality antenatal services for adolescent girls.

**Supplementary Information:**

The online version contains supplementary material available at 10.1186/s12978-021-01211-x.

## Plain language summary

Antenatal care is the routine health care of pregnant women in order to diagnose pregnancy complications and to provide information about lifestyle, pregnancy and delivery. Maternal deaths among teenage mothers in South Africa is high and is largely due to conditions that can be prevented or managed by high quality antenatal care. Timely and routine antenatal care is therefore crucial for pregnant teenagers. The way in which pregnant teenagers are treated by health care workers at antenatal clinics influences their clinic attendance. This study reports on the experiences of pregnant teenagers with health care workers, when accessing antenatal care in Cape Town, South Africa. Nineteen pregnant girls aged 13–19 years were interviewed. Some positive experiences such as respectful and supportive treatment were reported. However, more negative experiences were reported, including victimization; discrimination against being pregnant at a young age; feeling disregarded and excluded; a lack of information about pregnancy, health and childbirth; being discouraged from attending the clinics; and mental health distress. In conclusion, many teenagers felt mistreated and discriminated against by the health care workers, which discouraged their clinic attendance. Maternal health care workers in South Africa need to receive support and regular training to provide youth friendly antenatal care to teenage girls.

**Supplementary Information:**

The online version contains supplementary material available at 10.1186/s12978-021-01211-x.

## Introduction

Adolescent pregnancy is a serious public health concern in low- and middle-income countries, as it is associated with elevated risk of obstetric complications, gestational diabetes, anaemia, hypertensive disorders of pregnancy, and co-morbidities in comparison to adult women [1, 2]. Adolescent pregnancy is further associated with preterm delivery, low birth weight, infant mortality and respiratory diseases [1]. Mortality of mothers aged 15–19 years is estimated to be over a third higher than for women aged 20–24 years [3]. In addition, the mortality rate of infants born to 15–19 year old mothers is 50% higher than that of infants born to women aged 20–29 years [4].

In South Africa the adolescent fertility rate has declined from 72 births per 1000 girls aged 15–19 years old in 2012 to 68 births per 1000 girls in 2019 [5]. Despite the decline, this fertility rate is still over four times higher than the adolescent fertility rate in high-income countries [5]. The National Confidential Enquiries into Maternal Deaths (NCCEMD) in South Africa showed that the country’s institutional maternal mortality ratio (iMMR) in 2014–2016 was 76.9 deaths per 100,000 live births among adolescents [6]. Deaths among adolescent mothers accounted for 8.0% in 2011–2013 and 8.9% in 2014–2016 of all maternal deaths in the country [6, 7]. Over 71% of the deaths among teenage mothers in both reporting periods resulted from preventable factors including hypertension, non-pregnancy-related infections (HIV/AIDS-related, tuberculosis, or pneumonia), obstetric haemorrhage, and medical and surgical disorders [6, 7].

Timely and routine antenatal care for pregnant adolescents is crucial to reduce the risks associated with adolescent pregnancy. Timely and high-quality antenatal care enables the early screening, prevention, and management of risk factors for maternal mortality and poor health outcomes among adolescents. These risk factors include, for example, hypertension, tuberculosis, HIV, respiratory conditions, high blood sugar and anaemia. For example, timely initiation of antiretroviral therapy as a result of early antenatal booking results in reduced vertical HIV transmission from mother to baby [8, 9]. Antenatal care also provides an opportunity for health promotion and education on birth options, and a skilled health provider assisting with the delivery and infant care practises. The Guidelines for Maternity Care in South Africa recommend that women enrol in antenatal care in their first trimester (≤ 12 gestational weeks) [10], as this is an appropriate time for an obstetric assessment and early referral for management of identified risk factors [11]. However, only 53% of antenatal clinic bookings in South Africa occur before 20 weeks [12], but the rate of adolescents in these booking is unknown. Almost a quarter (23%) of adolescents do not attend the minimum requisite of at least four antenatal visits, which is similar to that of all pregnant women [13].

Previous studies have shown that timely health care seeking among pregnant women and adolescents is impacted largely by the manner in which they are treated by maternal HCWs [14]. Nurses and midwives are the point of contact in public health facilities and are the most common type of health care worker (HCW) encountered by women for sexual and reproductive healthcare (SRH). The negative attitudes and untoward behaviours of HCWs, such as rudeness, indifference, condescension, and dismissal, can alienate adolescents seeking SRH [14–19]. South African studies show that the negative treatment of adolescents by some HCWs is due to HCWs lacking the skills for adolescent SRH service provision and some HCWs having negative social norms towards adolescent sexual practices [20, 21]. HCWs have also been shown to disrespect the preferences and opinions of women for birthing options, and their confidentiality and privacy [17, 22]. The fear of being reprimanded and the assumption by HCWs that pregnant adolescents engage in promiscuity are barriers to accessing SRH services [22–24].

Adequate services to pregnant adolescent girls need to be provided by HCWs without limitations or prejudices [25]. HCWs need to demonstrate a positive and caring attitude, which results in the adolescent feeling welcomed into the facility [25]. The South African National Department of Health manages the implementation of the National Adolescent-Friendly Clinic Initiative (NAFCI), which uses an Adolescent and Youth Friendly Services (AYFS) approach. The initiative aimed to provide youth friendly services for adolescents that addressed barriers to service uptake, including training healthcare providers to deliver quality adolescent services, and providing well-equipped and easily accessible facilities [26, 27].

Adolescent’s first-hand reports of the way they are treated by staff at antenatal clinics can inform initiatives to improve antenatal care. Prior qualitative studies among adolescents have identified negative treatment by HCWs in South Africa, but many of these studies have focused broadly on SRH services such as contraceptive access or HIV-related healthcare [22, 24, 28]. Other studies on pregnant adolescents are relatively old, were conducted in different regions of the country and do not provide in-depth information on the nature of the HCW interactions [29–31]. This study therefore specifically investigates the perceived experiences of pregnant adolescents regarding how they were treated by HCWs at antenatal clinics in Cape Town, South Africa. In light of the AYFS implementation, it aims to (1) investigate whether the negative HCW-adolescent experiences reported in some studies continue to remain a problem for pregnant adolescents in this region of the country, (2) probes into the nature of the HCW interactions, and (3) investigates if negative HCW interactions continue to discourage adolescent antenatal attendance. The findings can be applied with a view to improving interventions and policies to enhance the provision of quality antenatal care to adolescents thereby increasing adolescent antenatal care-seeking behaviour. The current paper is part of a larger project that investigated pregnant adolescents’ general knowledge, attitudes, motivating factors, and experiences regarding antenatal appointment attendance and their healthcare behaviours during pregnancy.

## Methods

The study utilised a qualitative phenomenological approach and was conducted among pregnant adolescents who accessed antenatal care services in the public health sector. The study was conducted between May and August 2017. In a phenomenological study, data is obtained from people who have experienced a phenomenon to develop a composite description of what the participants experienced and how they experienced it [32]. The study observed the consolidated criteria for reporting qualitative research (COREQ): the 32-item checklist for interviews and focus groups [33].

### Participants and setting

The study was conducted in four of the eight health subdistricts in Cape Town, Western Cape Province, South Africa. The recruitment involved purposive selection of six public healthcare facilities within these subdistricts, that provide Basic Antenatal Care (BANC) services. The facilities were selected because (1) they had high numbers of young BANC attendees, and (2) the communities they serve collectively represented the various population groups, languages and socio-economic groups of public health BANC attendees in Cape Town. One facility was a public health clinic (PHC) and five were Midwife Obstetric Units (MOUs). All six facilities were in urban settings.

A study overview was presented to the facility managers, informing them about the study and inviting them to discussions about the strategies for recruitment and data collection with minimal disturbance to the facilities’ daily activities. For the recruitment process, English, Afrikaans and isiXhosa were chosen as the languages of communication when recruiting and interviewing participants, as these were the predominant official languages spoken in the chosen sub-districts in Cape Town.

Participants were purposively approached face-to-face and recruited from the BANC waiting areas at the selected facilities. The inclusion criteria for participants included age 13–19 years, currently pregnant, and English, isiXhosa or Afrikaans speaking. At each facility, researchers introduced the study in the respective languages to the BANC attendees in the waiting areas. Adolescents who indicated interest in participating in the study were directed to a researcher who explained the study to them individually. in their language of choice. The researcher assessed their language preference and their eligibility to be included in the study by asking questions to confirm their age, language preference and pregnancy status and to confirm that they were attending antenatal care. Over 25 adolescents were recruited, of which 19 consented to participation in the study. The contact information of the consenting participants was obtained to schedule focus group discussions with them at a later time.

### Ethical considerations

The study was part of a wider study that was approved by the Research Ethics Committee of the Human Sciences Research Council (REC 2/17/08/16). Thereafter, permission was sought from the Provincial Government of the Western Cape and Department of Health to conduct research within the communities’ public health clinics and MOUs. All participants received information sheets outlining the purpose, objectives, the benefits and potential risks of the study, as well as their privacy and confidentiality, and the right to withdraw from the study at any time. In addition to individual consent, participants under the age of 18 years required consent from a parent or guardian. Participants were also informed of and consented to dissemination of the study findings, through publications and reports, which included extracts of the qualitative transcripts without mentioning of names.

### Data collection

Data collection was conducted at the health care facilities, in a vacant room at a time convenient for all participants and in their language of choice. No one else was present in the data collection rooms besides the participants and the interviewer. Some participants reported difficulty in travelling to the facilities and in making time to attend focus group discussions ( FGDs), particularly those who attended school. This therefore determined the number of participants that could be made available to attend a FGD in a particular language. Kamberelis and Dimitriadis [34] endorsed the use of very small focus groups, as fewer participants can create an environment where participants feel more comfortable sharing their thoughts and experiences. The term ‘mini-focus groups’ refers to small FGDs of 3–6 people when participants have specialised experiences that can be discussed in a group [35–37]. In addition, Morgan (2012) advocates for 2- or 3- person groups [38]. Therefore, due to the logistical circumstances and the need for an intimate and confidential environment where adolescents can speak about their pregnancy experience in an interactive setting, two to five participants in a mini focus group (mini FGD) were acceptable. Four mini FGDs were held for 15 of the 19 participants. Two focus groups included Xhosa speaking participants and two included Afrikaans speaking participants. Four participants did not attend their scheduled mini FGDs due to logistical reasons. The research team were unable to group these participants into other mini FGDs, because they had different languages of choice; a common date and time could not be agreed upon for a scheduled mini FGD; and they were unable to travel to other mini FGD sites. These four participants expressed their interest in continuing with the study and expressed preference for individual interviews. They were therefore engaged in individual face to face in-depth interviews (IDIs) instead of the mini FGDs. The IDIs provided an opportunity to explore areas of discussion in more detail.

A topic guide with open-ended questions and probes was developed by the research team, and was used to guide the mini FGDs and IDIs. The questionnaire consisted of items on participants’ experiences and perceptions of antenatal clinic attendance and their experiences with the HCWs at the facilities. The questionnaire was pilot tested among two pregnant adolescents and their feedback was used to refine the questions. In addition, a brief questionnaire was used to obtain the demographic characteristics of the participants.

On average, the IDIs were approximately 60 min and the mini FGDs were approximately 120 min. Only one interview per participant or one mini-FDG per group was conducted and there were no repeat interviews or mini-FGDs. Prior to data collection, all participants were informed of and verbally consented to being audio-recorded. All recorded mini FGDs and IDIs were transcribed verbatim and translated. All research staff involved in the transcription and translation processes were trained on procedures for transcribing and translating qualitative data. To enhance the validity of the study, the researchers evaluated the transcripts and translation against the audio recordings and the identified themes. Field notes were taken during the recruitment and data collection to reflect on the researchers’ own thoughts and assumptions. Weekly meetings were held by the research team to discuss the field notes and experiences to ensure the interviews and mini focus groups covered the relevant aspects of antenatal care experiences. Data saturation was discussed after the third mini-FGD and second IDI. Data saturation had been reached by the fourth FGD and fourth IDI, as no new viewpoints had emerged.

Three researchers conducted the recruitment, facilitated the mini FGDs and conducted the IDIs. The researchers were all female, had master’s degrees in social psychology, were working as researchers on the wider study at the time of data collection and had prior experience in qualitative research. In addition, they had lived in communities similar to the participants and were bilingual (two in English-Afrikaans and one in English-IsiXhosa). The research assistants received training in qualitative interviewing techniques. There were no pre-existing relationships between the participants and the researchers prior to recruitment and data collection.

### Analysis

Thematic analysis was used to analyse the data based on the guidelines of Robson (2011) [39]. Two independent researchers (RS and DS) read the transcripts, coded the transcripts, and organised the codes into themes. Throughout the analysis process, the data sets and code extracts were crosschecked between RS and DS for verification. Atlas TI software was used to facilitate data management and analysis. The decision coding tree is presented in Additional file 1: Figure S1. Decision coding tree.

## Results

Of the 19 participants, 12 were isiXhosa speaking and were Black African and 7 were Afrikaans speaking and were of mixed race (“Coloured”).^1^ It must be noted that there was a limited number of very young adolescents aged 13–15 years at the facilities during the recruitment period. The mean age of the participants was 18.6 years. More than half of the participants reported being out of school (59%), more than a quarter were in school (26%) with the least number of participants attending university (16%). The majority (84%) of the participants were pregnant for the first time and 16% had a previous pregnancy.

Experiences with HCWs that would both reinforce and discourage antenatal attendance were found, with the discouraging experiences being reported far more frequently. The results are presented according to the following themes and sub-themes: Experiences that reinforce antenatal clinic attendance: 1. Respectful and supportive treatment; and Experiences that discourage antenatal clinic attendance: 1. Victimization, 2. Discrimination against being pregnant during the teenage years, 3. Experiencing disregard and exclusion, 4. Inadequate provision of information about pregnancy, health and childbirth, 5. Clinic attendance discouragement and alienation and 6. Mental health turmoil. Direct quotations are presented to support the description of the results for each theme.

### Reinforcing experiences

#### Respectful and supportive treatment

Some pregnant adolescents reported being treated with respect and kindness by some of the HCWs. They felt supported, comfortable and welcomed by these HCWs. They also reported appreciation for being able to ask questions and have information provided to them.

“there’s some nurses that’s nice to you and show you respect and they always helpful, talk to you, ask you questions …Treat you with love and respect. They made me feel welcome.”

**Participant IDI 4**

“When you tell them, you have a certain pain somewhere they tell you what is happening. Like the nurse that we were saying is nice, I really like that nurse”

**FGD 1, Participant 1**

“They will come and tell you ‘it’s just you and me, there’s no one here whatever you ask me will stay between us and if you don’t want your husband here then only me and you can chat’. They make you feel relaxed. They make you feel at home –where you can talk and they have been there. They are interactive with you”

**Participant IDI 4**

“It depends on the person! If the [nursing] sister looks like someone – she is friendly. Like the sister [nursing] who examined me, took my blood pressure, and such things. I asked her many questions!”

**Participant IDI 3**

### Discouraging experiences

#### Victimization

Many pregnant adolescents felt that they were treated unfairly when they visited the facilities and their visits felt unpleasant because the HCWs behaved more rudely and offensively towards them than towards older pregnant women. They indicated that due to their young age, HCWs saw them as immature, promiscuous and shameless. They felt that they were treated differently from their older counterparts. They reported that the treatment by the HCWs made them feel as though they were guilty of committing a crime or doing something seriously wrong. Participants felt that their reputation was being blemished and that the rudeness of the HCWs led the older pregnant women to gossip about them. The adolescents felt that the actions of the HCWs were demeaning and belittling, and alienated them, even though they were at the clinics for the very same treatment as the older pregnant women.

“They [health care workers] mistreat us; they shout at us and are very rude. …. But the other [nursing] sisters, their faces show ‘No, don’t ask me’ –unapproachable.”

**FGD 1, Participant 1**

“But what I don’t like about the clinic, is there’s people that is very rude. They’re very rude they don’t ask you; they just assume.”

**Participant IDI 4**

Rudeness by the HCWs was reported as frequent among the participants and the dissatisfaction with the relationship with HCWs could be noted in the tone in which the participants spoke. In addition, one narrated a patient-provider interaction that she had witnessed during one of her clinic visits, whereby she felt that the HCW was judgmental and embarrassing in the approach of a pregnant teenager:

“The girl that was with me was 15 or 16. She was very thin and you could see in her face she is young. Her mother came with her and then the mother went home because she was hungry. So, she told her [asked] mother if she can go home, she also wanted to leave the day hospital. So, the [nursing] sister said ‘why do you want to go with your mother, you did not sleep with your mother!’ La la la… the [nursing] sister scolded the girl. It is almost like because she is young. ‘There is a thing like a condom –Why don’t they use condoms?’”

**Participant IDI 3**

Another participant reported unpleasant encounters with HCWs, and she was rebuked when she requested a pregnancy test. The encounter below indicates a failure by the HCW to carry out the duties of a health professional in a professional and respectful manner:

“I started suspecting I was pregnant when I was a month far along, and then I went to the clinic to ask for a pregnancy test but the nurses told me to go buy myself a pregnancy test and bring it. When I told her that I saw the sign which clearly states that free pregnancy tests are offered at the clinic she shouted at me saying they don’t test for pregnancy so I just walked away because she was shouting at me in front of other patients and saying out loud everything I had come to do at the clinic”

**FGD 2, Participant 4**

### Discrimination against being pregnant during teenage period

According to the participants’, their age was a big issue of contention, as they felt stigmatized because of this. The participants felt that this led to the HCWs being particularly intolerant and impatient with them. Furthermore, they saw the differences in the attitudes and behaviours of the HCWs towards older pregnant women and themselves as younger women and did not understand why there was differentiation in treatment, or the harsh, judgmental attitude that they had to endure just because they were younger.

One participant said, “*with the nurses, if you are young, they are nasty to you*”.

The majority of the participants agreed that the HCWs did not have a right to be judgmental. Participants expressed concern about how a HCW would treat adolescents who fell pregnant due to being raped and who presented at facilities for healthcare along with the other pregnant adolescents. They were concerned that these adolescents may be humiliated by the HCWs without knowing the context of their situations and needs. Participants also reported that being in a relationship and becoming pregnant was not a reason for HCWs to treat them unfairly and negatively. Some participants felt that if a woman asked for contraceptives, the HCWs should have been happy that she was practicing safe sex. They preferred purchasing contraceptives from a pharmacy, where they were not judged, or they asked their partners to buy it for them.

The excerpts below demonstrate the stigmatizing treatment received when pregnant adolescents went to health care facilities:

“Just because I’m a teenager and pregnant –doesn’t mean you (health care workers) have to treat me, well, differently towards a married woman. It doesn’t mean that you have to. “oh, ok you are a teenager, come, come, come and this and that.” No. We all…I have the same…we all are pregnant you can’t treat me different you don’t know the reason, maybe I got raped and felt pregnant or maybe I was in a relationship and then fell pregnant. So, basically you can’t treat me differently”

**FGD 4, Participant 2**

“They say negative things about the fact that you are young and pregnant, they even go as far as telling others to look at you, saying “look at this one, pregnant at this age.” But the strange thing is that even when a young person goes to the clinic for contraceptives, they are rude and ask questions like “why do you wanna get contraceptives at such a young age”. Worst thing is one falls pregnant and they still give you a hard time!”

**FGD 1, Participant 1**

Another participant whose late arrival for her consultation resulted in her being shouted at when a nurse reportedly said to her:

“Why are you late, you fall pregnant at such a young age but can’t even do something as simple as getting here on time!”

**FGD 1, Participant 2**

### Experiencing disregard and exclusion

The participants also felt that they were neglected when they attend their clinic appointments, because they were not attended to on time, and the HCWs were unhelpful and made them feel unimportant. Some participants felt that the HCWs were possibly under pressure in their work, and their lack of understanding of pregnant adolescents led to their neglecting the needs of the adolescents.

“No, the nurses make you feel like you are wasting your time, they act like you have nothing better to do by making you wait there and not helping.”

**FGD 2, Participant 2**

“Sometimes they just go into that examination room and sit there and the worst thing about being at the clinics is that when it gets to tea or lunchtime they leave.”

**FGD 2, Participant 4**

“They wait for you to ask questions, and if you don’t then they don’t care”

**FGD 3, Participant 2**

“Basically, you just going to collect your tablets and they tick it off, they don’t still speak much to you.”

**FGD 3, Participant 1**

One participant expressed being ignored by administrative staff when she was referred to them to collect documentation:

“You can stand almost for how long and calling someone and no one is coming…they [administrative staff] just walk past you like you not even standing there.”

**IDI 4**

One participant resorted to using other resources as substitutes for antenatal clinic attendance, due to the neglectful behaviour of the HCWs, which deterred her from going to the healthcare facility.

“I’ve been at home for a long time, I just didn’t come to the clinic because the nurses are useless, they were not helping me, so I whenever there was something I wanted to know I would just log onto Facebook and find out.”

**FGD 2, Participant 2**

### Inadequate provision of information about pregnancy, health and childbirth

The adolescents reported that HCWs withheld information regarding the mothers’ and unborn children’s health and when asked specific questions they were unhelpful. Participants said that although very expensive, visiting a private doctor or another clinic further away that they perceived as being more youth-friendly, was a preferred choice as it gave them the information required and their questions were answered with clarity and care. All participants reported that they feared asking pregnancy related and general health questions as they often received negative responses resulting in them being humiliated and feeling disempowered, or that the HCWs displayed an unwillingness to respond to the questions. The narratives below also indicate that the young women were able to compare the services and assistance they were receiving in comparison to what they should have been receiving, thus seeking help from the internet and private physicians.

“Huh-uh I don’t yet have enough information. Okay, yes they gave us a list of things to pack for the baby. But how will I know how I must lay –I didn’t practice this process before!”

**Participant IDI 3**

“Like –if come here –there are times where they don’t tell you –like this is how far your baby is. Your baby is fine nothing like that. They do their tests; they don’t tell you like there’s no movement in the baby. Like if I go to the doctor [private] or something or for a test they will tell you like okay your baby is moving. That’s – the baby is on the right tract now. Then there’s just that people who don’t tell you at all. You are like ‘what’s happening, wondering’”

**Participant IDI 4**

“Look, I asked the [nursing] sister what they were doing, and she just shouted at me asking me why I wanted to know saying “you like to be forward, why you want to know everything”! So, I was never even able to ask for a test and didn’t even know whether I was tested or not.”

**FGD2, Participant 2**

“Not at all, because they (health care workers) are always rude or they tell you to go ask your parents, or even ask you, “When you were making the baby did you not know what would happen?”

**FGD 2, Participant 1**

“Some of the nurses are intimidating, because you just take one look at them and they seem so unapproachable that you feel scared to ask questions.”

**FGD 1, Participant 2**

When the participants were asked how they respond to a HCW who is perceived as unapproachable, one of them said “then I will rather not ask them any questions, or I will go and ask that other [nursing] sisters.”

**Participant IDI **

The nurse’s unwillingness to provide answers to their questions also resulted in a sense of helplessness and confusion as illustrated in the quote from another participant:

“They even tell you, you are too big for your shoes you should know, even the fact that you are pregnant means that you already know a lot, so I don’t know what they expect us to do or where they expect us to go when they tell us that we have information which we don’t.”

**FGD 2, Participant 2**

### Clinic attendance dissuasion and alienation

The participants’ experiences with HCWs greatly influenced their clinic attendance. A key finding was that the participants felt discouraged to seek antenatal care early in their pregnancy, or they discontinued their attendance because the quality of care did not meet their expectations and they wanted to avoid encountering the HCWs. Some even resorted to changing to a clinic further away in the hope of receiving better treatment and care.

“First of all, I don’t want to be there… Just that they think that you from [name of community], you are like [that community] and so they have to treat you like [the community]”

**FGD 4, Participant 2**

“It hurts a lot because when you come to the clinic you expect that you will get help and get support and advice so when these very people that you trust to give you information are rude to you it’s very discouraging.”

**FGD 2, Participant 1**

One participant confirmed her pregnancy early on, but only sought antenatal care a few months later due to an earlier encounter with a HCW who was rude:

“I left, went to buy my own pregnancy test and it came out positive. But I only went back to the clinic three months later to start receiving antenatal care.”

**FGD 2, Participant 4**

One of the girls had resolved to change clinics with hopes of getting better treatment:

“So that’s why I’m saying I’m going to start over from [Name of facility]. I’d rather start over.”

**FGD 2, Participant 2**

### Mental health turmoil

Participants recognized that although they were young, they had made the choice to keep the baby after birth as some of them had support from their families and partners. They felt that not providing the information and services for pregnancy and birthing needs forced some adolescents to consider abortion. They reported that abortion was a legitimate option for some adolescents to be away from the discriminating maternal health care environment. Due to the discrimination they faced some young women were in emotional turmoil and opted for the unsafe option of abortion by themselves.

“And the [nursing] sisters –it feels as though –especially with the young girls that are pregnant. They are discriminated against because we are pregnant. The nurses shout at us ‘you had to know you must not have sex, there is a thing like a condom now you sit with this thing!’ I told the one I’m studying sports management, so she said ‘now you sitting with a sport management. Do you know how it is to raise a child blah blah blah’? That also puts off the girls, the [nursing] sister shouting on their heads. Then they decide they’re going for an abortion. They don’t have time for nagging, that’s what aggravates me the most is the lots of tests they must do.”

**Participant, IDI 3**

Another participant mentioned that some adolescents opt for unsafe abortion as they *“abort the child themselves”*, and not in a hospital or clinic.

## Discussion

This study provides insight into pregnant adolescents’ experiences with HCWs when seeking antenatal care at public health facilities in Cape Town, South Africa. While both positive and negative interactions with HCWs were reported, the negative ones far outweighed the positive ones. A range of disconcerting HCW attitudes and behaviours were reported by the adolescent participants, which resulted in them feeling victimised, alienated and disillusioned, thereby influencing their satisfaction with care and attitudes to care seeking. Many participants perceived HCWs as rude, judgmental, neglectful, and discriminatory. Mistreatment and discriminatory behaviour by HCWs toward adolescents seeking antenatal care have been confirmed in other studies both in South Africa and internationally [14, 25, 31, 40–42]. Furthermore, a systematic review on attitudes and behaviours of maternal health care providers toward patients found that negative patient-provider interactions can impact on the patients’ emotional well-being, satisfaction with care, utilization of antenatal, delivery and postnatal services, and maternal health outcomes [14]. The mistreatment, even if experienced by only some adolescents, can perpetuate a perception of fear among their peer and community groups, resulting in adolescents being fearful of HCWs even before arriving at the clinic [40].

The adolescents perceived that the HCWs had prejudicial and stigmatizing attitudes regarding their being pregnant at a young age and having sex at a young age. HCWs’ prejudicial attitudes based on the adolescents’ appearances and the communities in which they live were also reported. In other sub-Saharan African countries, HCWs were found to have reservations towards provision of SRH services to adolescents, that were often linked to religious or cultural beliefs and attitudes toward premarital sex and childbearing, contraceptive use during adolescence and abortion [21, 25, 43]. Other studies found that groups perceived as ‘socially deviant’ including teenagers, unmarried women and those who present late for antenatal care experienced verbal abuse and neglectful treatment from HCWs [14].

Participants reported that they did not receive sufficient pregnancy and childbirth related information from HCWs, including what examinations were being conducted and why. Many were uncomfortable to ask for information, while some who did were rebuked. Among participants who had positive experiences with HCWs, a consistent finding was their feeling comfortable enough to ask questions and having access to information. Similarly, a lack of communication from HCWs to young pregnant women was reported in Zimbabwe, where the women were not given an opportunity to clarify doubts or ask questions about antenatal procedures, and the poor communication seemed to be linked to the young age of the women [44]. Given that pregnant adolescents have lower health literacy and little knowledge of what to expect during pregnancy, it is vital for HCWs to provide them with clear and comprehensive health education and create an enabling environment for them to ask questions or clarify doubts. Access to information increases adolescents’ autonomy in practising healthy behaviours. Due to the lack of information provided by HCWs, some participants sought help from private physicians, travelled further to other clinics that they perceived as being more youth-friendly or used the internet to guide them. These alternate options can create unnecessary financial costs, thus further perpetuating social disadvantage and alienation in this vulnerable group [45].

Several participants reported thoughts of abortion among pregnant adolescents in their communities, that they linked to feeling unsupported in the healthcare system. The multiple stressors including lack of preparedness for and information on pregnancy and child rearing, stigma from the community or school, family and partner pressures, and social and financial hardship can contribute to the decision-making process to terminate the pregnancy. These stressors compounded with a distressing healthcare environment can deter adolescents from seeking safe abortions at a clinic or hospital [46].

HCWs have an ethical and professional duty to promote dignity and to adopt anti-discriminatory behaviour towards patients [47]. Time pressures, staff shortages and lack of resources at facilities were important determinants of maternal HCWs’ service provision to adolescents in South Africa, in that maternal HCWs need to examine large numbers of women each day and therefore were unable to dedicate the requisite time and attention to each patient [21]. Similar studies attribute negative behaviours and attitudes of maternal HCWs to unfavourable working conditions and lack of training, resulting in stress, fatigue, and frustration [14]. Furthermore, some HCWs report lacking skills for adolescent SRH service provision and that their own norms and values towards adolescent sexual behaviours affect their service provision to this age group [21]. Due to their maturity levels, lack of knowledge and experience with pregnancy, childbirth and healthcare seeking, and stigma associated with adolescent pregnancy; adolescents’ may require greater sensitivity in their interactions with HCWs. Given that negative attitudes of HCWs are a reported barrier to adolescent SRH care seeking [14], adolescents may therefore be more susceptible to being discouraged from attending antenatal care than adult women as a result of negative HCW interactions.

A recent study assessing the South African National Department of Health’s Adolescent Youth Friendly Services (AYFS) programme (AYFS) provision identified the need for HCWs to receive regular training on how to administer youth friendly services [23]. HCWs need to have the appropriate skills for attending to the maternal healthcare needs of adolescents. This will increase their self-efficacy which is a strong predictor of the intention to provide maternal healthcare services to adolescents [20]. Currently, less than 45% of South African maternal HCWs receive annual continuous professional development (CPD) training in antenatal care and in implementing the national guidelines for child and maternal care services [48]. Therefore, nurses and midwives need to be mandated to attend CPD training each year in all the modules of maternal healthcare. They should receive training in administering youth friendly services and in the ethics of healthcare. HCWs need to adhere to the national guidelines for administering adolescent pregnancy services. Failure to adhere to these guidelines, for example denying a patient a pregnancy test, as reported by a participant in this study, constitutes a violation of the guidelines and dereliction of duty. Furthermore, regular evaluations of HCW service provision should be conducted. It would be beneficial for HCWs to undergo behavioural change interventions to change their attitudes and norms regarding pregnant adolescents. One such intervention is values clarification training, which allows HCWs to clarify their values and attitudes in the context of their work [49]. An evaluation of abortion values clarification workshops among health workers and community leaders in South Africa showed effectiveness in increasing knowledge about termination of pregnancy laws and changing attitudes and behaviours regarding reproductive choices [49]. At a health systems level, staff shortages need to be addressed to alleviate HCW time constraints. These measures can enhance the knowledge, attitudes and skills of HCWs to enable them to behave in a supportive and educative way toward adolescents, thereby providing quality antenatal care and improving antenatal clinic attendance.

The study has some limitations. Firstly, participants were not provided with the transcripts of the interviews and could not be contacted to be given an opportunity to provide feedback on the findings due to logistical reasons. Secondly, the study focused on facilities in urban settings in Cape Town, and therefore findings cannot be generalised to rural areas. Thirdly, due to availability and language preference of the participants, the focus groups were limited to very small sizes. In this instance, one-on-one interviews for all participants could have been more consistent. Lastly, the study did not interview or follow up participants who were attending clinics but subsequently stopped. Data on antenatal care experiences between adults and adolescents is lacking. Therefore, despite its focus on adolescents, the study would have benefitted from including adult pregnant women for comparison. Additionally, a mixed methods approach as well as interviews with the health professionals working in the facilities would have helped to better contextualize the findings. Despite this limitation, the findings contribute to the body of knowledge on the quality of care received by pregnant adolescents at antenatal clinics in urban South Africa and can be used to inform strategies to improve antenatal care for adolescents.

## Conclusion

More negative than positive experiences between pregnant adolescents and HCWS were reported, suggesting an overall discordant patient-provider relationship. Adolescents felt disregarded and disrespected by HCWs and felt stigmatized because of their age. They also reported a lack of information on pregnancy because they felt unable to clarify doubts or ask questions. The negative attitudes of the HCWs influenced their satisfaction with the antenatal care services and their healthcare seeking decisions. The findings suggest that adolescent and youth friendly antenatal care should be prioritized in South African public health facilities. HCWs need to exercise patience, encouragement and tolerance, so that a balanced patient-provider relationship is facilitated, thereby improving timely antenatal clinic attendance.

## Supplementary Information

**Additional file 1: **Figure S1. Decision coding tree.

## Data Availability

The data used in the current study is available from the corresponding author on reasonable request.

## Abbreviations

HCW: Health care worker
SRH: Sexual and reproductive healthcare
BANC: Basic Antenatal Care services
MOUs: Midwife Obstetric Units
FGD: Focus group discussion
IDI: In-depth interview
AYFS: Adolescent Youth Friendly Services
CPD: Continuous professional development

## Footnotes

1 Population groups were reported as per Statistics South Africa’s standard population groups. Statistics South Africa (2020). South Africa Mid-year Population Estimates 2020. Pretoria: Stats SA. Available at http://www.statssa.gov.za/?page_id=1854&PPN=P0302.
