# Supplementary material for: A mixed reception: perceptions of pregnant adolescents’ experiences with health care workers in Cape Town, South Africa
Source: Reprod Health. 2021 Aug 4;18:167. doi: 10.1186/s12978-021-01211-x (PMC8336349; doi:10.1186/s12978-021-01211-x)
Supplement: Supplementary file 1 — Additional file 1: Figure S1. Decision coding tree. [file 12978_2021_1211_MOESM1_ESM.docx]

Figure S1. Decision coding tree. HCW: Health care worker
